# ClimateEU, scale-free climate normals, historical time series, and future projections for Europe

**DOI:** 10.1038/s41597-020-00763-0

**Published:** 2020-12-04

**Authors:** Maurizio Marchi, Dante Castellanos-Acuña, Andreas Hamann, Tongli Wang, Duncan Ray, Annette Menzel

**Affiliations:** 1 CNR - Institute of Biosciences and BioResources (IBBR), Florence division, Via Madonna del Piano 10, I-50019 Sesto Fiorentino (Florence), Italy; 2 Department of Renewable Resources, University of Alberta, 751 General Services Building, Edmonton, AB T6G 2H1 Canada; 3 Centre for Forest Conservation Genetics, Department of Forest and Conservation Sciences, University of British Columbia, Vancouver, BC V6T 1Z4 Canada; 4 Forest Research, Northern Research Station, Roslin, Midlothian, Scotland United Kingdom; 5 Department of Ecology and Ecosystem Management, Technical University of Munich, 85354 Freising, Germany

**Keywords:** Atmospheric dynamics, Climate and Earth system modelling

## Abstract

Interpolated climate data have become essential for regional or local climate change impact assessments and the development of climate change adaptation strategies. Here, we contribute an accessible, comprehensive database of interpolated climate data for Europe that includes monthly, annual, decadal, and 30-year normal climate data for the last 119 years (1901 to 2019) as well as multi-model CMIP5 climate change projections for the 21^st^ century. The database also includes variables relevant for ecological research and infrastructure planning, comprising more than 20,000 climate grids that can be queried with a provided *ClimateEU* software package. In addition, 1 km and 2.5 km resolution gridded data generated by the software are available for download. The quality of *ClimateEU* estimates was evaluated against weather station data for a representative subset of climate variables. Dynamic environmental lapse rate algorithms employed by the software to generate scale-free climate variables for specific locations lead to improvements of 10 to 50% in accuracy compared to gridded data. We conclude with a discussion of applications and limitations of this database.

## Abstract (summary)

**Table:** 

Measurement(s)	temperature of air • volume of hydrological precipitation
Technology Type(s)	digital curation • computational modeling technique
Factor Type(s)	monthly temperature measurement • monthly precipitation measurement
Sample Characteristic - Environment	climate system
Sample Characteristic - Location	Europe

Machine-accessible metadata file describing the reported data: 10.6084/m9.figshare.13190834

## Background & Summary

Interpolated climate data have become an essential tool for researchers, natural resource managers, policy makers and analysts to assess climate change impacts and to develop climate change adaptation strategies^1–3^. Applications in research, natural resource management and infrastructure planning usually require long-term climate baseline data with appropriate spatial and temporal resolution (often 30-year climate normal periods), records of past climate variability (historical time series data) and future predictions from Atmosphere-Ocean General Circulation Models (AOGCMs). However, such data are not readily available in consistent formats and require expert knowledge to efficiently subset, overlay, resample, reproject, or query to obtain climate estimates for locations or regions of interest^4^. There are some notable on-line resources that provide some of this functionality (e.g., www.worldclim.org, https://www.ncdc.noaa.gov, https://daymet.ornl.gov, www.prism.oregonstate.edu, http://www.climatewizard.org), but these resources do not simultaneously provide raw data access and powerful query tools for researchers, while maintaining ease of access and convenient data extraction and visualization tools for resource managers and policy makers.

We have previously developed databases with software front-ends for North America that build on quality datasets generated by various research groups^5–8^. These original datasets are further enhanced by our software packages by using lapse rate adjustments that dynamically vary for each variable and each geographic location^9^, adjusting for the difference between the grid elevation and the elevation of the location of interest (i.e. obtained from a GPS position for a sample location, or through an elevation estimate from a high resolution digital elevation model). Previous work^9–11^ showed that this scale-free approach to estimating climate variables for specific locations significantly improved the statistical accuracy of climate estimates compared to the original grids. The European environment is amongst the most studied in many research fields, from agriculture to biology and forestry^12,13^, and a similar software for this region of the world was missing. The *ClimateEU* software we are presenting here aims to fill this gap.

Here, we aggregate and enhance several high-quality, publicly available databases that are integrated with a free, easy-to-use software frontend. This software package and database can be obtained from the figshare repository^14^ with the latest version also available via anonymous download at http://tinyurl.com/ClimateEU. Our objective is to provide a comprehensive solution to: (1) generate climate grids for a wide range of climate variables and time periods at any resolution and in any projection for custom study areas; (2) accurately characterize climate conditions for a location and time period of interest, such as a study site, for which an on-site station data is not available or for which the station record is incomplete; (3) generate historical time series for one or many sample locations for time series analysis; and (4) provide simple access to future projections from 15 selected AOGCMs for climate change impact and adaptation planning that relies on comparing past 30-year climate normal conditions with those that may be expected over the coming decades.

Another unique characteristic of our software solution is that the size of the total database of more than 20,000 climate surfaces is kept manageable by storing only the 1961–1990 climate reference period at high resolution while expressing all other periods (historical monthly, seasonal, annual or decadal as well as future projections) as anomalies from this reference normal at lower resolution^15,16^. The statistical accuracy of climate estimates is partially recovered by overlaying the coarse resolution anomaly grids on the high-resolution reference baseline^15^.

For continental-scale studies that cover Europe, we also provide a useful subset of 5,200 grids at 1 km and 2.5 km resolution in Albers equal area projection. These grids include 48 monthly variables (monthly precipitation, minimum, maximum, and average temperature), as well as 36 bioclimatic variables that are relevant in ecology, agriculture, or infrastructure planning (e.g. growing and chilling degree days, heating and cooling degree days, Hargreave’s moisture deficit and reference evaporation, beginning and end of the frost-free period, etc.). This paper describes the database in detail and discusses the strengths and limitations of this database for use by natural resource managers, policy makers and researchers. We further evaluated a representative subset of climate variables against observed station data, and we report error estimates for climate normals and historical data.

## Methods

### Baseline climate period 1961–1990

*ClimateEU* uses climate data for the normal period 1961–1990 as a baseline (or reference) dataset, which consists of 36 gridded data layers of monthly minimum temperature, maximum temperature, and precipitation, as well as an average elevation for each grid cell. The baseline dataset was compiled from different sources, resampled, and aggregated to a common 2.5 arcmin grid (approximately 5 km) and adjusted to 1961–1990 normal period. The sources include WorldClim v1.4^5^ for the temperature grids, an unpublished dataset developed with PRISM methodology^7,14^ for the European Alps region, provided Chris Daly and Manfred Schwaab, and ANUSplin interpolations for precipitation for the remainder of Europe.

The original data sources were developed at different resolutions for different time periods, and were therefore modified before aggregation into the *ClimateEU* data package. WorldClim v1.4 data was developed for the 1950–2000 period at 30 arcsec resolution, and was therefore adjusted to match the 1961–1990 normal period of the other datasets. To implement the adjustment, we first developed anomaly layers for 1950–2000 period relative to 1961–1990 using CRU data (described below), and used bilinearly interpolation to match this anomaly layer to the WorldClim v1.4 dataset. The anomalies were then subtracted from the 1950–2000 data and aggregated to the same 2.5 arcmin grid.

For the European Alps region, we used precipitation layers developed with PRISM methodology because this method is specifically designed to model rain shadows and orographic precipitation in mountainous terrain. PRISM-based interpolations cover the European Alps region between 43°–49°N latitude and 2°–17°E longitude. Precipitation grids for the remainder of Europe were developed using the smoothing spline software ANUSplin^17^ to interpolate station observations. The PRISM dataset for the European Alps region was seamlessly integrated by including the peripheral grid cells of the PRISM dataset in the training data for ANUSplin interpolation.

We use the standard normal period from 1961–1990 as baseline data as it presents several advantages. Spatial climate station coverage is excellent for this period and allows for the development of a reliable interpolated baseline data. The 1961–1990 period represents climate conditions at the start of a major anthropogenic warming signal. A period of global dimming due to particulate and sulphate pollution in the 1950s to 1980s may have masked a small anthropogenic warming signal in this period^18^. Also, the period from 1961 to 1990 has been used as a reference period for long-term climate change assessments by the World Meteorological Organisation.

### Historical climate estimates 1901–2019

Monthly historical data from 1901 to 2019 are based on interpolated time series grids, CRU-TS 4.04, developed by Harris *et al*.^19^ at 0.5 degrees resolution (approximately 50 km). In order to overlay CRU-TS 4.04 historical data on our 1961–1990 baseline dataset, described in the previous section, we first calculated a CRU-TS 4.04 average for the same 1961–1990 time period. By subtracting the CRU-TS 1961–1990 average from individual years and months, we derived monthly CRU anomaly surfaces (deviations from the 1961–1990 normals). These anomalies were downscaled to 2.5 arcmin using bilinear interpolation and overlaid on *ClimateEU* baseline grids. This anomaly approach, also referred to as change factor or delta method^20^ preserves spatial variation due to topographic effects (temperature gradients along elevation gradients, or orographic precipitation on windward facing slopes or rain shadows on leeward slopes of mountain ranges), without the need to model these effects at high resolution for individual years and months of the entire historical period from 1901 to 2019.

### CMIP5-based future projections

For future projections, we applied the same anomaly approach, as described above for historical data, to the CMIP5 multimodel dataset corresponding to the IPCC Assessment Report 5^21^. We summarized annual AOGCM projections into 30-year time periods, hereafter referred to as 2020 s (2011–2040), 2050 s (2041–2070), and 2080 s (2071–2100). As AOGCMs data are available at various spatial resolutions, ranging from 0.75 × 0.75° through 2.85 × 2.85°, we interpolated the grids to a common resolution of 1 × 1° using bilinear interpolation for simpler integration into the *ClimateEU* software package. This common resolution was chosen as to not lose spatial information for most AOGCM models. To apply the delta method, we converted AOGCM projections to anomalies for each normal period by subtracting their 1961–1990 predictions.

We selected 15 AOGCMs to represent all major clusters of similar AOGCMs by Knutti *et al*.^22^. Within clusters, we selected models that had the highest validation statistics in their CMIP3 equivalents, leading to the following selection: CanESM2, ACCESS1.0, IPSL-CM5A-MR, MIROC5, MPI-ESM-LR, CCSM4, HadGEM2-ES, CNRM-CM5, CSIRO Mk 3.6, GFDL-CM3, INM-CM4, MRI-CGCM3, MIROC-ESM, CESM1-CAM5, GISS-E2R. For each of these models, we selected two Representative Concentration Pathways (RCPs) scenarios with a radiative forcing value of + 4.5 and + 8.5 W/m^2^ expected in 2100 relative to pre-industrial values. For the chosen AOGCMs, these forcing values would result in projected global warming increase (and ranges) of approximately + 1.4 °C ( ± 0.5) by the 2050 s and + 1.8 °C ( ± 0.7) by the 2080 s for RCP4.5. For RCP8.5, the average global climate change projections for mean annual temperature would be + 2.0 °C ( ± 0.6) by the 2050 s and + 3.7 °C ( ± 0.9) by the 2080 s. We aggregated all available individual runs from each of 15 AOGCMs × three future normal periods (2020 s, 2050 s, 2080 s) × two RCPs (4.5 and 8.5) to arrive at 90 future projections that can be used to assess uncertainty in the near, medium, and long term. Additionally, we derived average ensemble projections across all 15 models listed above.

### Scale-free dynamic downscaling

The main feature of the *ClimateEU* software tool presented here is the ability to apply a dynamic downscaling approach to the gridded baseline data to generate scale-free climate data for any location in Europe between 34.26°and 71.24° degrees latitude and –10 .74° and 44.24° longitude. The system works using lapse-rate based elevation adjustments that vary with each variable, elevation, and geographic location, providing a set of 84 scale-free climatic variables (annual, seasonal or monthly). The empirical local lapse rates were described as polynomial functions for each monthly temperature variable based on geographic coordinates (latitude, longitude, and elevation). Taking the partial derivative of the function with respect to elevation, the rate of change in a variable in response to a change in elevation is obtained for any given latitude, longitude, and elevation^23^.

The elevation adjustment for a requested location or grid cell is calculated with these partial differential equations by the *ClimateEU* software on demand. Normally, a point of interest queried by a user has a different elevation value than the elevation of the corresponding *ClimateEU* grid cell. The partial differential equations are then applied to the elevation difference between a grid cell elevation and the elevation of a location of interest. After elevation adjustments had been carried out for monthly variables, the full set of biologically relevant climate variables were either calculated (seasonal and annual summaries) or estimated (e.g. growing degree days, frost free period) using a correlative approach with values derived from daily weather station data (see Wang *et al*.^9^ for details).

### Data quality assessments

Data quality assessments were carried out by comparing the observations from weather stations with climate variables generated by the *ClimateEU* software for station locations. We assume that most or all of the climate station data that we use for quality assessments^8,24,25^ were also used for the development of interpolated climate grids (PRISM, CRU-TS, WorldClim, ANUSplin). Therefore, our quality assessment is not an independent validation using withheld data. Therefore, in addition to evaluating the *ClimateEU* estimates directly to observations from stations, we also compared the *ClimateEU* output against the baseline dataset to evaluate the benefits of the environmental lapse rate adjustments and the effectiveness of delta downscaling approach.

**Fig. 1:**
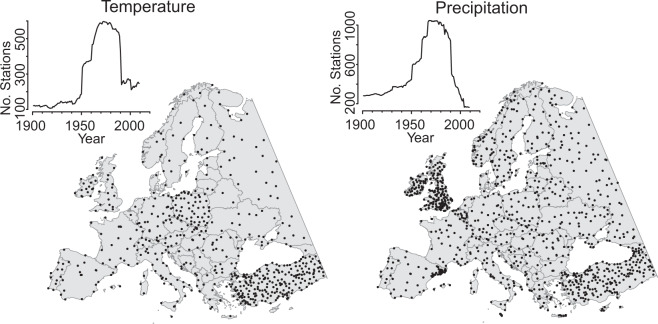
European climate stations used for quality assessments of gridded data and historical time series from the *ClimateEU* software package, which carries out data overlays and environmental lapse rate adjustments for spatial downscaling.

Prior to the quality assessment, we combined and filtered the climate station databases, retaining only stations with records that exceeded 30 years, and with less than 5% missing values. We also applied a spatial filter, removing nearby duplicate stations by retaining only the highest quality station record in a 20 arcmin grid cell and a 100 m elevation interval (Fig. 1); see Castellanos-Acuña and Hamann^26^ for details on the quality assessment and filtering procedures. For these stations, we evaluated how well historical estimates from *ClimateEU* account for variance explained in original climate station data, measured as the R^2^ of a simple linear model between predicted and observed climate value. To provide another metric describing the magnitude of errors in units of °C and millimeters precipitation, we report Mean Absolute Error (MAE), i.e. the mean absolute difference between *ClimateEU* estimates and observed station data.

## Data Records

We make our climate datasets available in two formats, the *ClimateEU* software package and climate grids. The *ClimateEU* software package includes the climate grids of the 1961–1990 normal period, historical and future anomalies, performs local downscaling, and generates many climate variables on demand for any location of interest (single or multiple). It can also process custom digital elevation models (DEMs) at any resolution or geographic projection with an input file in text format (.csv or.txt). In total, approximately 20,000 data layers that represent different variables for different historical and future time periods can be queried with the software package. The *ClimateEU* software package can be obtained from the figshare repository^14^, with the latest version also available via anonymous download without registration requirements at http://tinyurl.com/ClimateEU. The current version 4.71 includes monthly, annual, decadal, and 30-year normal climate data for the last 119 years (1901 to 2019), as well as multi-model CMIP5 climate change projections for the 21^st^ century.

**Fig. 2:**
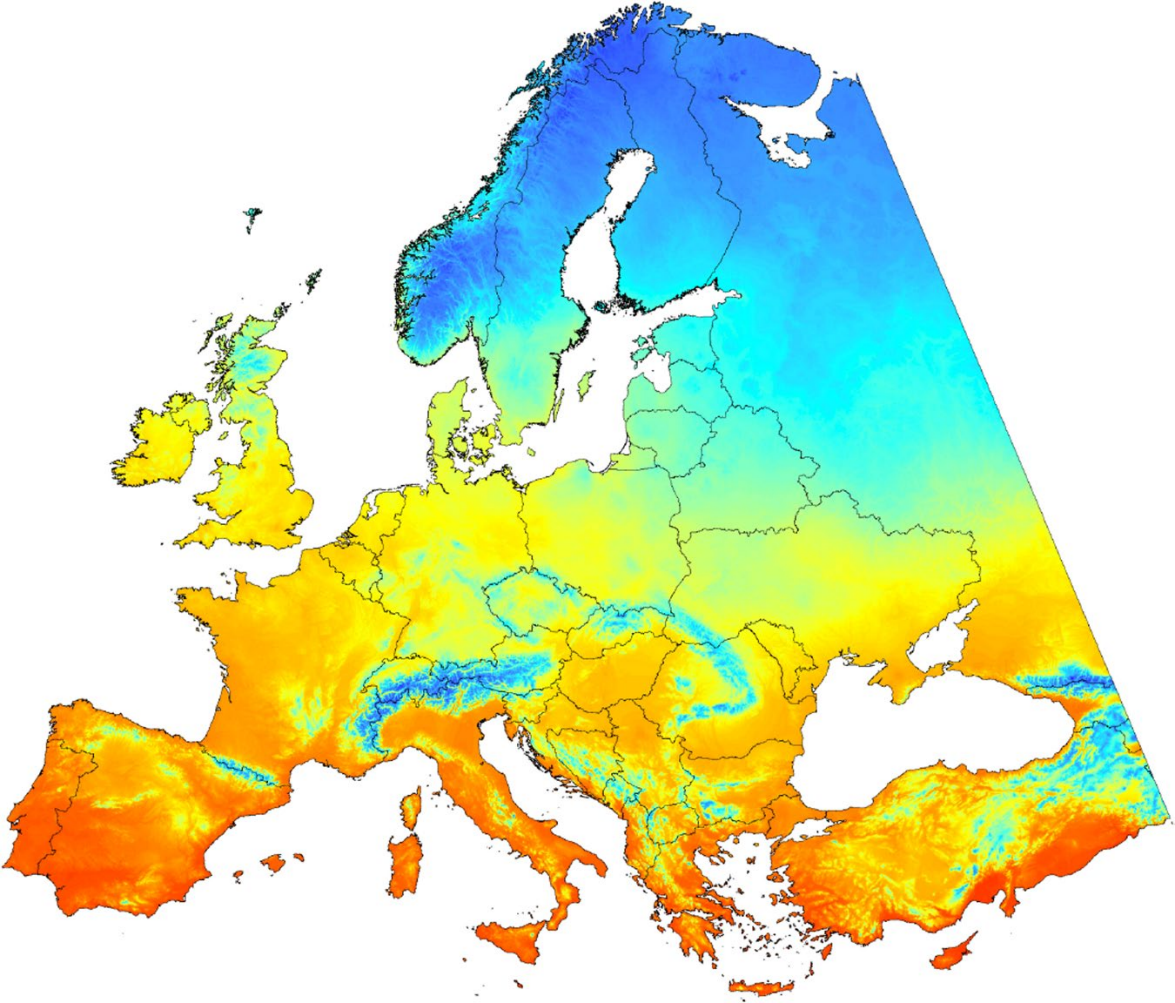
Example climate grid for mean annual temperature, showing the extent of gridded climate surfaces for Europe, west of 44°E latitude at 1 km resolution in Albers equal area projection. A set of 4,800 grids are available at http://tinyurl.com/ClimateEU, comprising monthly and bioclimatic variables for historical periods and future projections.

Secondly, we provide climate grids generated by the *ClimateEU* software package. The figshare data repository^14^ includes a set of 5,200 climate grids at 1 km and 2.5 km resolution for all of Europe west of 44°E longitude in Albers equal area projection (Fig. 2). The latest versions of these grids can also be downloaded at http://tinyurl.com/ClimateEU. The grids are available for a 1961–1990 normal reference period and various future projections from AOGCMs. Climate variables include 48 monthly variables for precipitation, maximum temperature, minimum temperature and average temperature for 12 months the year, as well as 36 bioclimatic variables.

The bioclimatic variables provided in both the software package and climate grids include mean annual temperature (MAT), seasonal means for precipitation, maximum temperature, minimum temperature and average temperature (e.g. PrecDJF, PrecMAM, etc.), mean warmest month temperature (MWMT), mean coldest month temperature (MCMT), temperature difference (TD = MWMT-MCMT), mean annual precipitation (MAP), mean growing season (May to September) precipitation (MSP), annual heat:moisture index calculated as (AHM = MAT + 10)/(MAP/ 1000)), summer heat:moisture index (SHM = (MWMT)/(MSP/1000)), degree-days below 0 °C or chilling degree-days (DD < 0), degree-days above 5 °C or growing degree-days (DD > 5), degree-days below 18 °C or heating degree-days (DD < 18), degree-days above 18 °C or cooling degree-days (DD > 18), the number of frost-free days (NFFD), frost-free period (FFP), the Julian date on which FFP begins (bFFP), the Julian date on which FFP ends (eFFP), precipitation as snow between August of the previous year and July of the current year (PAS), extreme minimum temperature over 30 years (EMT), Hargreave’s reference evaporation (Eref), and Hargreave’s climatic moisture deficit (CMD).

## Technical Validation

### Evaluation of historical time series data

**Fig. 3:**
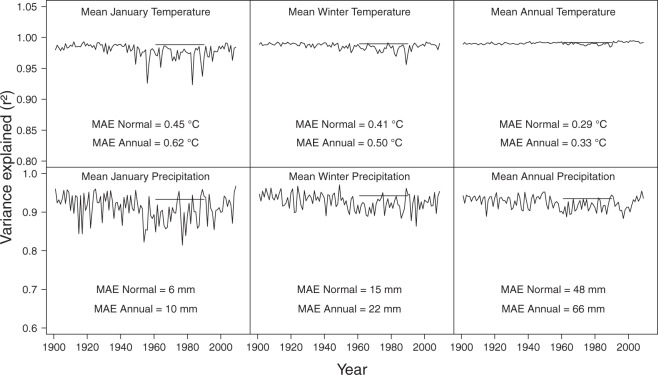
Evaluation of historical estimates from *ClimateEU* showing the variance explained in original climate station data over time for two monthly, two seasonal, and two annual climate variables. The horizontal bars represent the R² values for the 1961–1990 normal estimates. In addition, the Mean Absolute Errors (MAE) represents another metric describing the magnitude of errors in units of °C and millimeters precipitation, i.e. the average absolute difference between *ClimateEU* estimates from observed station data.

Climate variable estimates for individual years generated by the *ClimateEU* software package for the historical period from 1901 to 2019 generally compare favorably with the 1961–1990 normal average (Fig. 3). This validates the anomaly approach, where we only store the 1961–1990 baseline grids at high resolution, and historical data is reconstructed by overlaying 0.5° resolution anomaly grids to estimate historical data for individual years. Both metrics that we use for quality evaluations are generally high, with variance explained in climate station data largely above 0.9. Mean absolute errors were mostly below 0.5 °C for temperature variables and less than 10 mm for monthly precipitation variables, although there was more variation among individual yearsfor precipitation (Fig. 3). Estimates increase in precision for longer seasonal and annual periods. Note that the errors for precipitation sums over longer periods need to be evaluated relative to the larger precipitation values. In general, precision of climate estimates increases with the length of time that the variables represent, i.e., mean annual temperature estimates are more precise than seasonal variables, which are in turn are more precise than monthly variables. Also, estimates of precipitation variables are generally less precise than estimates for temperature variables.

Monthly historical climate layers from the CRU-TS dataset were developed at a 0.5° grid size, corresponding to approximately 50 km resolution. Climate variables at this relatively coarse resolution are expected to be less accurate, especially in mountainous terrain. However, by converting the CRU-TS climate estimates to anomalies and downscaled the *ClimateEU* software package, historical data can be generated with precision comparable to to high-resolution climate normal layers (Fig. 3, compare monthly estimates with 1961–1990 normal indicated by bar). Storing only one high resolution baseline climate grid, and 119 annual low resolution anomaly layers (1901–2019) reduces the total database size by two orders of magnitude, with minimal sacrifice to spatial resolution in complex terrain.

### Effectiveness of lapse rate adjustments

Breaking down the evaluation for climate stations located below and above 1,000 m reveals the difficulty of estimating climate variables in mountainous terrain and the effectiveness of the *ClimateEU* algorithms in carrying out environmental lapse-rate adjustments (Table 1). Mean absolute errors of climate estimates are highest for climate values directly obtained from a grid for montane areas (Table 1, lower left quarter), often exceeding 1 °C. In contrast, unadjusted climate values obtained directly from gridded data for stations below 1000 m elevation are more precise with MAE values around 0.5 °C (Table 1, upper right quarter).

**Table 1:** Data quality assessment of the *ClimateEU* 1961–1990 baseline dataset with and without lapse-rate based elevation adjustments, based on mean absolute errors (MAE) between weather station data and interpolated grids.

Variable	*ClimateEU* - elevation adjusted			Without adjustment		
	Monthly	Seasonal	Annual	Monthly	Seasonal	Annual
**Stations** <**1000 m**						
Tmin (°C)	0.52	0.48	0.44	0.64	0.61	0.60
Tmax (°C)	0.41	0.37	0.33	0.53	0.49	0.46
Tave (°C)	0.36	0.33	0.26	0.46	0.44	0.38
Prec (mm)	5.0	13.0	44.0	5.0	13.0	44.0
**Stations > = 1000 m**						
Tmin (°C)	0.78	0.75	0.71	0.92	0.89	0.81
Tmax (°C)	0.57	0.53	0.45	1.01	0.98	0.94
Tave (°C)	0.58	0.54	0.44	1.29	1.27	1.21
Prec (mm)	8.3	23.3	81.1	8.0	22.6	78.0

The statistics without adjustment refer to climate values for weather station locations directly extracted from the 1961–1990 baseline grid. In addition, the *ClimateEU* software can carry out lapse-rate based adjustment based on the elevation difference of the climate grid cell versus the recorded elevation of the climate station.

Lapse rate adjustments for temperature variables substantially improve the quality of climate estimates for montane regions, resulting in MAE values ranging from 0.5 °C to 0.8 °C. The adjustment is most effective for maximum and average temperature estimates, while improvements are relatively minor for minimum temperature estimates (Table 1, lower left quarter). The reduced improvement for minimum temperature was also found in other regions of the world^10,27^. We suspect that the reason for the limited effectiveness of environmental lapse rate adjustments for monthly minimum temperatures in mountainous terrain is caused by temperature inversions that make it difficult to derive lapse rate adjustments that generally apply for a geographic area. Lapse-rate adjustments are less important for stations located below 1,000 elevation but still yield approximately a 20% reduction in error for temperature variables (Table 1, lower left quarter).

Error estimate for precipitation variables suggests that also these variables are more difficult to estimate in mountainous terrain, with mean absolute errors almost twice as large for weather station locations above 1,000 m than those located below (Table 1). Since variation in precipitation is generally smaller across an altitudinal range than temperature and does not vary linearly^7,28,29^, the *ClimateEU* software does not carry out lapse rate adjustments. For this reason, the errors of *ClimateEU* estimates are identical or near-identical to estimates when querying gridded climate data directly. For estimates of precipitation variables at high resolution, the *ClimateEU* software will carry out a simple bilinear interpolation for mapping and display purposes, so that the resulting grids do not show tile artifacts of the underlying coarser original climate grids. However, this interpolation does not result in any improvements in precision. In fact, for mountainous areas the estimates are very slightly inferior compared to the original gridded data, about 3% increase in mean absolute errors (Table 1).

The second metric that we use for evaluation, Variance explained (R²) also shows that climate variables in mountainous terrain are more difficult to estimate (Table 2). The metric also allows for a direct comparison of temperature and precipitation estimates. For locations below 1,000 m, there is a clear difference in quality of monthly precipitation (R² of 0.94) and temperature estimates (R² around 0.98). However, in mountainous areas the quality of precipitation estimates is comparable to estimates of minimum monthly temperature, both with relatively low R² values around 0.85. Non-adjusted average temperature (Tave) and maximum temperature (Tmax) values obtained directly from grids have the lowest R² values, dropping below 0.8 (Table 2, lower right quarter). That said, after lapse-rate adjustment, Tave and Tmax estimates are substantially improved, Tmin values show only minor improvements (Table 2, lower left quarter).

**Table 2:** Data quality assessment of the *ClimateEU* 1961–1990 baseline dataset with and without lapse-rate based elevation adjustments, based on variance explained (R^2^) in climate station data by estimates from interpolated grids.

Variable	*ClimateEU* - elevation adjusted			Without adjustment		
	Monthly	Seasonal	Annual	Monthly	Seasonal	Annual
**Stations** <**1000 m**						
Tmin	0.97	0.98	0.98	0.96	0.97	0.97
Tmax	0.99	0.99	0.99	0.98	0.98	0.99
Tave	0.99	0.99	0.99	0.98	0.98	0.98
Prec	0.94	0.94	0.94	0.94	0.94	0.94
**Stations** > = **1000** **m**						
Tmin	0.84	0.84	0.83	0.79	0.78	0.78
Tmax	0.90	0.90	0.90	0.76	0.75	0.72
Tave	0.97	0.97	0.98	0.86	0.87	0.86
Prec	0.84	0.84	0.87	0.85	0.85	0.89

The statistics without adjustment refer to climate values for weather station locations directly extracted from the 1961–1990 baseline grid. In addition, the *ClimateEU* software can carry out lapse-rate based adjustment based on the elevation difference of the climate grid cell versus the recorded elevation of the climate station.

Breaking down the evaluation further to individual months, for climate stations located in mountainous terrain, reveals that lapse-rate adjustments are most effective during the summer months. The largest errors for data obtained directly from original climate grids are for average monthly temperature between April and August, with error values around 1.4 °C (Table 3 left side), and here the lapse-rate elevation adjustments by the *ClimateEU* software are also most effective, reducing errors by 70%. The adjustments are least effective for minimum temperatures in winter with error reductions around 10%, again pointing to temperature inversions that are more frequent in mountainous terrain in winter, compromising the generality of empirical environmental lapse rate calculations.

**Table 3:** Mean Absolute Error (MAE) of estimates from climate grids, and changes in MAE broken down by month and variable after lapse-rate adjustment with the *ClimateEU* software, based on the elevation difference of the climate grid cell versus the recorded climate station.

Month	MAE without adjustment			Change in MAE due to adjustment		
	Tmin (°C)	Tmax (°C)	Tave (°C)	Tmin (°C)	Tmax (°C)	Tave (°C)
Jan	0.93	0.94	1.10	−0.04	−0.44	−0.59
Feb	0.93	1.00	1.28	−0.05	−0.55	−0.78
Mar	0.84	1.01	1.39	−0.12	−0.71	−0.93
Apr	0.88	1.05	1.44	−0.20	−0.99	−0.97
May	0.88	1.04	1.40	−0.18	−0.91	−0.98
Jun	0.92	1.05	1.41	−0.19	−0.87	−0.97
Jul	0.93	1.04	1.36	−0.16	−0.78	−0.93
Aug	1.01	1.08	1.37	−0.19	−0.79	−0.93
Sep	1.02	0.99	1.28	−0.14	−0.75	−0.83
Oct	0.95	1.10	1.26	−0.19	−0.75	−0.78
Nov	0.83	0.89	1.15	−0.11	−0.58	−0.67
Dec	0.86	0.89	1.07	−0.08	−0.48	−0.60

The evaluation was restricted to stations above 1000 m as the lapse-rate adjustment is primarily expected to yield benefits in mountainous regions.

## Usage Notes

The *ClimateEU* software package we provide is based on an equivalent methodology that were previously developed for North America, where our data products are widely used in engineering applications, environmental impact assessments, natural resource management, research in historical ecology, conservation biology, tree ring research, and climate change impact assessments. Compared to our North American datasets, the MAE and R² metrics for the European dataset are generally better, reflecting the higher density and longer history of climate station records. A selection of these applications have been reviewed by Mbogga *et al*.^15^ and Wang *et al*.^10,11^. Nevertheless, users of our climate databases should keep in mind that climatic features such as rain shadows or temperature inversions are modeled at a scale of kilometers, suitable to broadly represent mountainous terrain. Lapse-rate driven differences in temperature related variables along elevation gradients are accurately represented at a much finer scale, informative at a resolution of hundreds of meters. We should note, however, that all interpolated climate surfaces of this dataset are ultimately based on standard climate stations and consequently, microclimates that are driven by vegetation, water bodies, or topography at a scale of tens of meters are not are not represented.

Although the software described in this paper could be further enhanced by statistical downscaling to daily temporal resolution, our contribution does not provide climate variables with a temporal resolution of less than one month. Rather, our software and datasets are optimized for applications that require high spatial resolution (i.e. resolutions of hundreds of meters to a few kilometers). At those spatial scales, the simulation of weather events at a daily scale, or the estimation of the probability of weather events is not reliable enough (i.e. those estimates would hardly vary among adjacent cells). Therefore, our lowest temporal resolutions for historical data are monthly precipitation sums and temperature averages. For future projections, we only provide very low temporal resolution averages for 30-year normal periods (2011–2040, 2041–2070, and 2071–2100). High-resolution spatial data with coarse temporal scales still have a wide range of applications, particularly in climate niche or bioclimatic envelope modeling^30–32^. For example, in mountainous areas species communities can change in response to temperature gradients along elevation gradients at scales of hundreds of meters, and orographic precipitation and rain shadows drive local climatology at the scale of a few kilometers.

## Data Availability

The code used to carry out lapse-rate based elevation adjustments is publicly available under a Creative Commons Attribution 4.0 International license (CC BY 4.0). The *ClimateEU* software package can be obtained from the figshare repository^14^, with the latest version of this software and gidded database also available via anonymous download without registration requirements at http://tinyurl.com/ClimateEU.
